# Modern Contraceptive Utilization and Its Associated Factors among Indigenous and Nonindigenous Married Women of Reproductive Age Group in Jigjiga Town, Eastern Ethiopia, 2018

**DOI:** 10.1155/2020/6878075

**Published:** 2020-05-24

**Authors:** Hagos Degefa Hidru, Tariku Dingeta, Bezatu Menigiste, Berhe Etsay, Haftay Gebremedhin, Meresa Berwo, Guesh Gebreayezgi Asefa

**Affiliations:** ^1^Department of Public Health, College of Medicine and Health Sciences, Adigrat University, Adigrat, Ethiopia; ^2^School of Public Health, College of Medicine and Health Sciences, Haramaya University, Haramaya, Ethiopia; ^3^Department of Midwifery, College of Medicine and Health Sciences, Adigrat University, Adigrat, Ethiopia; ^4^School of Public Health, Department of Epidemiology and Biostatistics, Aksum University, Aksum, Ethiopia

## Abstract

**Background:**

The use of birth control and its promotion has potential benefits of reducing poverty, maternal, and child mortality. However, there is limited evidence regarding modern contraceptive utilization among indigenous and nonindigenous married women of the reproductive age group in Ethiopia. Therefore, this study aimed to assess the magnitude of modern contraceptive utilization and its associated factors among indigenous and nonindigenous married women in Eastern Ethiopia. *Methodology*. A community based comparative cross-sectional study design was conducted among married women of the reproductive age group from February 01 to March 01/2018 in Jigjiga town, Eastern Ethiopia. A total of 1004 married women were selected using a simple random sampling method. The collected data were entered into EpiData software version 3.1 and exported to SPSS version 22.0 for analysis. Bivariable and multivariable logistic regression analyses were used to identify the associated factors. Statistical significance was declared using 95% confidence interval and *p* value of less than 0.05.

**Result:**

A total of 987 study participants were included in the study with a response rate of 98.3%. The overall prevalence of modern contraceptive utilization among married reproductive age group women was found to be 19.9% (95% CI (17.4%-22.3%) with 26.5% and 11.4% for nonindigenous and indigenous women, respectively. Primary level of education (AOR 0.84; 95% CI (0.15-0.85) *p* 0.0001) and Somali in ethnicity (AOR 0.75; 95% CI (0.15-0.94) *p* 0.03) were associated factors among indigenous group, while age group 30-34 years (AOR 1.56; 95% CI (1.31-9.52) *p* 0.02) and being a housewife in occupation (AOR 0.49; 95% CI (0.42-0.96) *p* 0.04) were the associated factors among the nonindigenous group.

**Conclusion:**

Overall utilization of modern contraceptives was approximately one-fifth, with markedly lower use among indigenous participants. One-third of nonindigenous and less than one-seventh of indigenous were utilizing modern contraceptive methods. There is a need to further explore and understand the factors across indigenous and nonindigenous women for the use of modern contraceptives; hence, a longitudinal study is desirable.

## 1. Introduction

Family planning refers to intention planning of when to have children through the use of birth control measures. It means the use of contraceptives or other steps that allows individuals and couples to anticipate and have their desired number of children as well as to achieve healthy spacing and timing of their birth. Family planning has the potential benefits of reducing poverty, maternal, and child mortality that is occupying a critical role in the health and development of a population [1–3].

Globally, many obstacles remain to ensuring that women of reproductive age realize their right to modern contraception and reproductive health [4, 5]. Despite their desire to avoid or delay pregnancy, according to UNFPA 2010, around 215 million women in developing countries rely on traditional methods only, which have a high failure rate, or did not use any contraceptive methods at all. Given this unaddressed need for modern contraception, over 300,000 women die as a result of maternal or pregnancy-related complications each year [6, 7].

Globally, contraceptives help to prevent an estimated 2.7 million infant deaths and the loss of 60 million of healthy life in a year. At least 22,500 women died from unsafe abortion complications, 74 million unplanned pregnancies occur every year in the developing world [8, 9]. Avoiding different factors associated with the use of contraceptive methods could avert globally 54 million unintended pregnancies (80 per 1,000 women in Africa), 79,000 maternal deaths and one million infant deaths each year [10]. Though contraceptive utilization has a comprehensive benefit for women, it was one of underutilized public intervention in the least developed countries particularly low in Africa (33%), in Central and Southeast Asia with only 47%, whereas in Ethiopia, according to EDHS 2016 report, only 35% are using a modern contraceptive method [11, 12].

Also besides, the region has very low modern contraceptive coverage; according to EDHS 2016 report, it accounts for around 1.5% coverage as compared to other regions 56% in Addis Ababa, 47% in the Amhara region [6, 13]. However, it is of great interest to fill evidence-based the gap of why poor modern contraceptive utilization practices myriad obstacles as a result of factors such as lack of information, inability to take family decisions on their own, religions, desire number of family size, distance from health facilities, fear of side effects, and long waiting hour at the same time inadequate knowledge among indigenous and nonindigenous married women's of reproductive age group was assessed. Therefore, this study aimed to assess the magnitude of modern contraceptive utilization and its associated factor among indigenous and nonindigenous married women of reproductive age group.

## 2. Materials and Methods

### 2.1. Study Design and Period

A community based comparative cross-sectional study design was conducted among indigenous and nonindigenous married women of the reproductive age group in Jigjiga town, Eastern Ethiopia, from February 01 to March 01/2018.

### 2.2. Sample Size Determination and Sampling Technique

**Figure 1:**
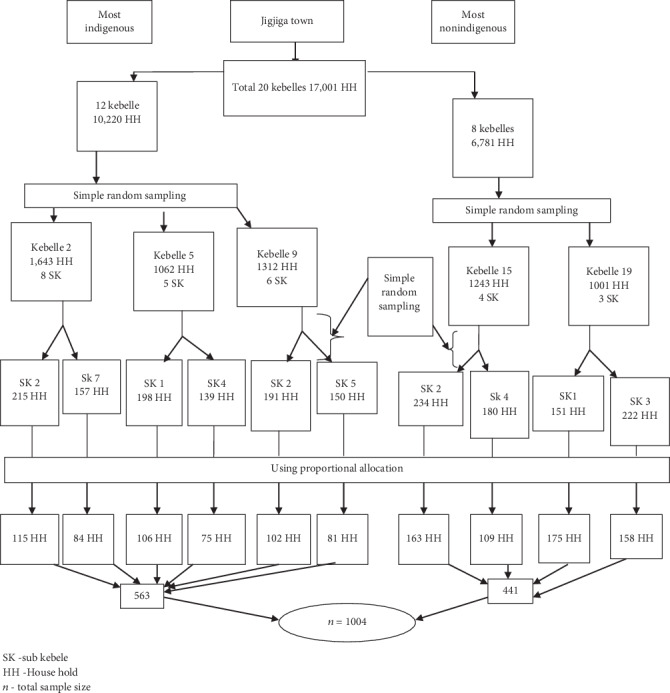
Schematic presentation of the sampling procedure of indigenous and nonindigenous married reproductive age group women in Jigjiga Town, Eastern Ethiopia, February1-March 1/2018.

The sample size was determined using a single population proportion formula by considering a confidence level of 95% = 1.96, the margin of error = 0.04, and *p* = 0.50 for both groups, because there are no studies done before on the region like this comparative among indigenous and nonindigenous groups. The calculated sample size was 280 for nonindigenous and 357 for indigenous, respectively, by using their target population 1037 (nonindigenous) and 5097 (indigenous) and by considering the 5% nonresponse rate and a design effect of 1.5, the final sample size was 1004 (441 nonindigenous and 563 indigenous). The multistage cluster sampling technique was used to reach a household level. A total of 1004 study participants were proportionally allocated based on the households from each sub kebeles, and then, a simple random sampling technique was used to identify study participants (Figure 1).

### 2.3. Data Collection Method

The data was collected through face-to-face interviewer-administered questionnaire. After extensive revision of the English questionnaire, the final English version was translated into local language by language expertise. The principal investigator trained the data collectors and supervisor for five consecutive days on instruction for the method; how to take informed written consent, how to approach participants, ethical procedure, and general information on modern contraceptive and the objective of the study. Eight university student fluent local language speakers (Somali and Amharic) and one supervisor were recruited.

### 2.4. Data Quality Control

The questionnaire was pretested on 5% of the study population in the nonselected kebeles to ensure clarity, wordings, logical sequence, and skip patterns of the questions. Appropriate modifications were made after discussing with the supervisor and data collectors before starting the actual data collection process. Every day the filled questionnaire was checked before a respondent goes from the setting by data collectors and supervisor.

### 2.5. Data Processing and Analysis

The completed data was entered using Epi-Data statistical software version 3.1 and then exported to SPSS version 22 for final analysis. Frequencies and percentages were used to present categorical data. The household wealth index was also constructed by using Principal Component Analysis (PCA). Odds ratios (ORs), 95% confidence intervals (CIs), and *p* value were calculated using a logistic regression model to determine association levels of predictors to the outcome variables. All variables with *p* value ≤0.15 were taken into the multivariable model to control for all possible confounders, and the variables were selected by all methods. A multivariable logistic regression analysis was used to estimate the adjusted OR of predictors to modern contraceptive utilization by controlling confounding factors. A variable having *p* < 0.05 was considered a statistically significant variable in all models. Before the inclusion of predictors to the final logistic regression models, the multicollinearity was checked using VIF < 10, tolerance tests >0.1, and standard error. The goodness of fit of the final logistic model was tested using Hosmer and Lemeshow test at a value of >0.05.

### 2.6. Operational Definitions

Nonindigenous women: married women of reproductive age group (15-49 years) who were not born in the region but currently native and living in the region for more than six months [14].

Indigenous women: married women of reproductive age group (15-49 years) were native, i.e., women who were born and living in the region irrespective of their ethnicity [14].

Utilization: use of any modern contraceptive method to space child and to protect unwanted pregnancy during the age of 15-49 years [15].

Modern contraceptive: refers to methods of child spacing or birth control other than natural methods like pill, Norplant, injectable, and IUCD [14].

Good knowledge: those women who scored points above or equal to the mean of the knowledge related questions on modern contraceptive utilization; those women who had scored points below the mean value were considered as poor knowledge.

## 3. Result

### 3.1. Socio-Demographic Characteristics of Study Participant

A total of 987 study participants were included in the study with a response rate of 98.3%. The mean age of nonindigenous and indigenous women was (29.6 + 8.3) and (28.8 + 7.5) years, respectively. More than one-fourth of the respondents 160 (28.8%) were in the age group of 20-34, and more than one-fifth of the respondents 106 (24.5%) were 25-29 age group for indigenous and nonindigenous, respectively. Considering the religion, 509 (91.7%) of the indigenous and 200 (46.3%) of nonindigenous were Muslim followers (Table 1). Concerning their live pattern 482 (78.8%) of indigenous and 370 (96%) of nonindigenous respondents were currently living with their husbands. Three hundred fifty-one (63.2%) indigenous and 357 (82.6%) nonindigenous respondents stated that their husband had no other wife. Five hundred two (90.5%) of indigenous and 380 (87.9%) nonindigenous respondents had children.

### 3.2. Contraceptive Knowledge

**Figure 2:**
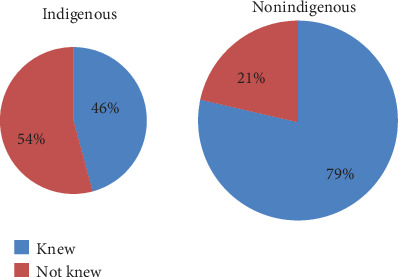
Frequency of indigenous and nonindigenous married reproductive age group women knowing any type of modern contraceptive in Jigjiga Town, Eastern Ethiopia, February1-March 1/2018.

From the total, 137 (68.8%) of indigenous and 129 (68.3%) of nonindigenous respondents had good knowledge. A total of 153 (60%) indigenous and 127 (37%) nonindigenous respondents knew the injectable type of contraceptive method (Figure 2). Two hundred sixty-two (47.2%) of indigenous and 278 (64.4%) of nonindigenous respondents knew at least one importance of modern contraceptive. More than two-thirds of the indigenous and nonindigenous groups had known the importance of contraceptive as pregnancy prevention. A total of 303 (54.6%) and 303 (70.1%) indigenous and nonindigenous respondents, respectively, had heard about modern contraceptive, and more than half of these respondents, 177 (58%) of indigenous and 170 (56%) of nonindigenous groups, were heard from the health professional.

### 3.3. Factors Related to Utilization of Modern Contraceptive Method

A total of 98 (17.7%) indigenous and 230 (53.2%) nonindigenous respondents had discussed modern contraceptive utilization with their husbands. Those experiencing waiting hour to get reproductive health service, 105 (52%) of indigenous and 323 (94%) of nonindigenous respondents were feeling short waiting hour, respectively.

**Figure 3:**
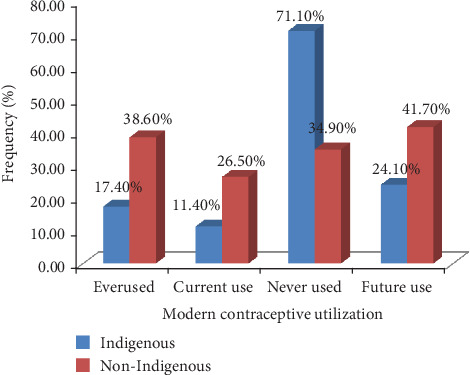
Frequency of indigenous and nonindigenous married reproductive age group women on utilization modern contraceptive in Jigjiga Town, Eastern Ethiopia, February1-March 1/2018.

Regarding the utilization of modern contraceptives only, 97 (17.40%) of indigenous and 167 (38.60%) of nonindigenous women have ever used any type of modern contraceptive. Of these reported users, 81 (83.5%) indigenous and 127 (76%) nonindigenous respondents have used the injectable method of contraceptive with more than half of indigenous 77 (55.8%) and nonindigenous 116 (55.5%) women receiving this method from nearby health posts. Sixty-three (11.4%) of indigenous and 114 (26.50%) of nonindigenous were current modern contraceptive users. From the current user, 45 (71.4%) and 71 (62.3%) of indigenous and nonindigenous received their contraceptives from local health posts (Figure 3).

### 3.4. Factors Significantly Associated with Modern Contraceptive Utilization

Nonindigenous women with the age group of 20-24 years [(AOR = 2.32, 95% CI: (1.65, 8.94)] and 30-34 years [(AOR = 1.56, 95% CI: (1.31, 9.52)] were 2.32 and 1.56 times more likely, respectively, to utilized modern contraceptives compared to age group 45-49 years. Those who had no formal education [(AOR = 0.74, 95% CI: (0.27, 0.96)] were 26% less likely to utilize modern contraceptive as compared to those with secondary and higher educational status. Housewives [(AOR = 0.49, 95% CI: (0.42, 0.96)] were 51% less likely to utilized modern contraceptive as compared to government employees. Women who had never used contraceptives because of the desire number of children [(AOR = 0.75, 95% CI: (0.52, 0.94)] were 25% less likely to utilize modern contraceptive as compared to those who had lack of knowledge (Table 2).

Indigenous women ages 20-24 years [(AOR = 2.87, 95% CI: (1.7, 9.20)] were 2.87 times more likely to utilize modern contraceptives as those in the age group of 45-49 years. Women with no formal education [(AOR = 0.34, 95% CI: (0.56, 0.96)] and primary level of education [(AOR = 0.84, 95% CI: 0.84 (0.15, 0.85)] were 66% and 16% less likely to utilize modern contraceptive, respectively, as compared to secondary and above educational status. Being a housewife in occupation [(AOR = 0.23, 95% CI: (0.17, 0.76)] was 77% less likely to utilize modern contraceptive than a governmental employer. Those of Somali ethnicity [(AOR = 0.75, 95% CI: (0.15, 0.94)] were 25% less likely to utilize modern contraceptives as compared to their Amhara counterpart. Those experiencing waiting times of less than one hour to get reproductive health services [(AOR = 2.21, 95% CI: (2.51, 9.53)] were 2.21 times more likely utilized modern contraceptive as compared to those waiting time greater than one hour. For women who have never used because of the desired number of children [(AOR = 0.65, 95% CI: (0.20, 0.95)] and for those whose religious disallows contraception [(AOR = 0.52, 95% CI: (0.12, 0.82)], they were 35% and 48% less likely to utilize modern contraceptive as compared to those who lacked knowledge (Table 2).

## 4. Discussion

In this study, the overall prevalence of modern contraceptive utilization among married reproductive age group of women was 19.9%; 95% CI (17.4%-22.5%) with 26.50%; 95% CI: (20.6%-31.8%) for nonindigenous and 11.4%; 95% CI (8.6%-13.9%) for indigenous participants. The difference among indigenous and nonindigenous might be due to those indigenous women who are more desired to children, because they are encouraged by their religious beliefs and lack of education, and information towards modern contraceptive as compared to the nonindigenous group. The findings of our results are lower when compared with a study done in Mexico, 58.3% of indigenous and 73.5% of nonindigenous [16]. The findings of this study were also lower than a study conducted in Tanzania (32.9%), Holeta Town (69.1%), and SNNPR (53.3%) [13, 17, 18]. This variance might relate to the differences in awareness, knowledge on importance of modern contraceptive utilization, desired number of children, a sample, and the period of the study.

The findings of the study about husband approval to utilize were similar to a study done in Bale Zone, Nigeria, Wolaita, and Nekmet women who were discussed with their husbands and approved to use modern contraceptives [3, 15, 19, 20]. Another study done in Gambela found the prevalence of 11.5% which is similar to our study findings, whereas a study done in Bangladesh among indigenous women showed 25.1% prevalence. Such difference might be attributed to differences in accessibility, service utilization, and knowledge towards modern contraceptive utilization in the current study [14, 21]. A study done in Gambela among nonindigenous found 36.4% prevalence which might reflect the difference in access to information, religion, fear of side effects, desired number of children, and lack of awareness on the importance of modern contraceptive utilization in the current study [21]. Studies done in Nigeria and Nekemet found that women with no formal education were less likely to utilize modern contraceptive which is similar to the findings of this study [15, 20]. Other studies done in Bahirdar, Gambella, and Ghana found women 20-24 years were more likely to utilize modern contraceptives than other age groups, which again is a similar finding to the current study [13, 14, 22].

### 4.1. Limitations

Since the study was cross-sectional, it might not show the temporal relation between the independent and dependent variables. The information collected was mainly through interviews, especially data collection tools for modern contraceptive utilization based on recall from the time marriage to the collection date. There may be a possibility that some of the responses might suffer from recall bias which may affect the results of the studies.

## 5. Conclusion and Recommendation

Generally, the overall utilization of modern contraceptives was approximately one-fifth, with markedly lower use among indigenous participants. One-third of nonindigenous and less than one-seventh of indigenous were utilizing modern contraceptive methods. Factors associated with utilization were belonging to the age group of 20-24 years, having no formal education, being a housewife, approved by husband, and having never used because of the desired number of children in both indigenous and nonindigenous groups. Being between 30 and 34 years was an associated factor among nonindigenous women, while the primary level of education, being Somali in ethnicity, and waiting time were the associated factors of indigenous respondents.

There is a need to further explore and understand the factors across indigenous and nonindigenous women for the use of modern contraceptives; hence, a longitudinal study is desirable. Further, it is important to share this information and evidence with decision makers and health care workers in order to address issues such as knowledge deficits on the use of modern contraceptions to inform programming and services offered.

## Figures and Tables

**Table 1:** Socio-demographic characteristics of married reproductive age group women of indigenous and nonindigenous groups in Jigjiga Town, Eastern Ethiopia, February1-March1/2018.

Variables		Indigenous (*N* = 555)		Nonindigenous (*N* = 432)	
		Frequency	Percent	Frequency	Percent
Education	No formal education	190	34.2	160	37
	Primary level	100	18	72	16.6
	Secondary and above	265	47.8	200	46.4

Occupation	Government employee	83	15.0	60	13.9
	Private employee	68	12.3	56	13.0
	Merchant	143	25.8	122	28.2
	Unemployed	65	11.7	47	10.9
	Housewife	144	25.9	105	24.3
	Student	52	9.4	42	9.7

Ethnicity	Somali	452	81.4	28	6.5
	Amhara	71	12.8	191	44
	Oromo	21	3.8	44	10.2
	Tigray	6	1.1	80	18.5
	Gurage	5	0.9	65	15
	Wolayta			24	5.8

Religion	Muslim	509	91.7	200	46.3
	Orthodox	40	7.2	188	43.5
	Protestant	6	1.1	44	10.2

Age group	15-19	20	3.6	3	0.7
	20-24	131	23.6	95	22.5
	25-29	127	22.9	79	18.7
	30-34	83	15	69	16.3
	35-39	130	23.4	105	24.8
	40-44	50	9	47	11
	45-49	14	2.5	25	6

**Table 2:** Factors associated with modern contraceptive utilization among indigenous and nonindigenous married reproductive age group women in Jigjiga Town of Eastern Ethiopia, February1-March1/2018.

Independent variables	Indigenous		COR (95% CI) *p* value	AOR (95% CI) *p* value	Nonindigenous		COR(95% CI) *p* value	AOR (95% CI) *p* value
					Current utilization			
	Current utilization							
	Yes	No			Yes	No		
Age group								
20-24	17 (12.9%)	114 (87.1%)	2.53 (0.74, 8.97) 0.05	2.87 (1.7, 9.20) 0.03	35 (36.8%)	60 (63.2%)	2.66 (0.95, 6.78) 0.02	2.32 (1.65, 8.94) 0.001
30-34	23 (24.7%)	70 (75.3%)	2.32 (0.83, 6.55) 0.06	2.09 (0.92, 8.30) 0.09	28 (35.4%)	51 (64.6%)	2.12 (0.74, 3.45) 0.05	1.56 (1.31, 9.52) 0.04
45-49	1 (7%)	13 (93%)		1.00	4 (16%)	21 (84%)		1.00
Educational level								
No formal education	14 (13.6%)	176 (86.4%)	0.11 (0.4, 0.85) 0.04	0.34 (0.56, 0.96) 0.02	44 (27.5%)	116 (72.5%)	0.37 (0.16, 0.84)0.02	0.74 (0.27, 0.96) 0.03
Primary level	9 (9%)	91 (91%)	0.28 (0.21, 0.79) 0.02	0.84 (0.15, 0.85) 0.0001	43 (45.7%)	51 (54.3%)	0.25 (0.12, 0.49) 0.6	0.37 (0.95, 1.5)
Secondary level and above	40 (15%)	225 (85%)		1.00	68 (34%)	132 (66%)		1.00
Occupation								
Housewife	12 (8.3%)	132 (91.7%)	0.14 (0.52, 0.92) 0.06	0.23 (0.17, 0.76) 0.05	29 (27.6%)	76 (72.4%)	0.51 (0.24, 0.92) 0.05	0.49 (0.42, 0.96) 0.04
Private employee	4 (5.9%)	64 (94.1%)	0.24 (0.12, 0.49) 0.07	0.63 (0.48, 1.05)	15 (27%)	41 (73%)	0.43 (0.18, 0.92) 0.08	1.51 (0.83, 6.22)
Government employee	26 (31.3%)	57 (68.7%)		1.00	25 (41.6%)	35 (58.4%)		1.00
Husband								
Approved	45 (40.2%)	67 (59.8%)	1.99 (1.05, 3.78) 0.001	2.17 (1.37, 8.44) 0.02	80 (29%)	194 (71%)	1.56 (0.38, 0.95) 0.04	2.46 (1.04, 7.49) 0.001
Disapprove	5 (38.5%)	8 (61.5%)		1.00	25 (16%)	132 (84%)		1.00
Reason for never using contraception								
Religious does not allow	10 (9.3%)	98 (90.7%)	0.16 (0.25, 0.89) 0.02	0.52 (0.12, 0. 82) 0.03	7 (23%)	23 (77%)	0.29 (0.38, 2.45)	1.3 (0.54, 9.35)
Desired number of children	12 (10%)	109 (90%)	0.14 (0.13, 0.95) 0.002	0.65 (0.20, 0.95) 0.0001	11 (28%)	29 (72%)	0.26 (0.31, 0.64) 0.02	0.75 (0.52, 0.94) 0.04
Lack of knowledge	12 (11.8%)	90 (88.2%)		1.00	5 (22%)	14 (78%)		1.00
Ethnicity								
Somali	36 (17.9%)	416 (82.1%)	0.59 (0.29, 1.17) 0.02	0.75 (0.15, 0.94) 0.03	15 (39%)	23 (61%)		1.09 (0.96,7.23)
Amhara	13 (18.3%)	58 (81.7%)		1.00	109 (43%)	145 (57%)		1.00
Overused contraceptives								
Yes	22 (15.8%)	117 (84.2%)	1.72 (0.98, 4.72) 0.06	1.08 (1.21, 10.53) 0.02	95 (33.7%)	187 (66.3%)		2.75 (1.25, 9.32) 0.05
No	41 (9.9%)	375 (90.1%)		1.00	60 (33.5%)	119 (66.5%)		1.00
Waiting time/hour								
Less than one hour	51 (48.6%)	54 (51.4%)	1.95 (1.76, 9.63)	2.21 (2.51, 9.53) 0.04	75 (46.6%)	86 (53.4%)		1.2 (0.5, 2.8)
Greater than one hour	12 (12.4%)	85 (87.6%)		1.00	80 (29%)	195 (71%)		1.00
Wealth index								
Low incomes	28 (13.9%)	174 (86.1%)		1.00	95 (50.3%)	94 (49.7%)		1.00
High income	35 (19%)	149 (81%)	1.93 (0.79, 3.75) 0.07	2.31 (0.85, 3.45)	60 (35%)	110 (65%)	0.53 (0.27, 0.71) 0.06	0.45 (0.56, 2.04)
Reasons for MCM know								
Space number children	10 (9%)	102 (91%)		1.00	26 (43%)	35 (57%)		1.00
Prevention of pregnancy	39 (14%)	250 (86%)	2.16 (1.08, 6.45) 0.08	1.05 (0.96, 10.6)	88 (40%)	131 (60%)	3.06 (1.98, 4.72) 0.07	3.n (1.38, 4.34) 0.001

CI: confidence interval; COR: crude odds ratio: AOR: adjusted odds ratio; MCM: modern contraception method.

## Data Availability

The data used to support the findings of this study are available from the corresponding author upon request.
